# The Influence of Basic Psychological Needs and Passion in Promoting Elite Young Football Players’ Development

**DOI:** 10.3389/fpsyg.2020.570584

**Published:** 2020-11-16

**Authors:** José L. Chamorro, Rubén Moreno, Tomás García-Calvo, Miquel Torregrossa

**Affiliations:** ^1^ Departamento de Ciencias del Deporte, Faculty of Sport Sciences, Universidad Europea de Madrid, Madrid, Spain; ^2^ Department of Didactics of Musical, Plastic and Corporal Expression, Faculty of Sport Sciences, University of Extremadura, Cáceres, Spain; ^3^ Departamento de Psicología Básica, Evolutiva y de la Educación, Faculty of Psychology, Universitat Autónoma de Barcelona, Barcelona, Spain; ^4^ Sport Research Institute, Universitat Autónoma de Barcelona, Barcelona, Spain

**Keywords:** self-determination theory, motivation, self-regulation, concentration disruption, reflective thinking, dualistic model of passion

## Abstract

Motivational variables and cognitive skills have been identified as important in an athlete’s development. The aim of this study is to explore the influence of each basic psychological needs satisfaction on player’s development regarding reflection and concentration disruption with the mediation of types of passion in Spanish young elite football players. A total of 487 elite U18 male football players (*M_age_* = 17.43, *SD* = 0.71) completed measures of basic psychological needs satisfaction, passion for football, reflective thinking, and concentration disruption. Measurement models were defined using exploratory structural equation models. The results provide support for the model, where each psychological needs satisfaction prompted reflection and had a negative influence on concentration disruption with the mediation of harmonious passion. In addition, obsessive passion mediated the positive relationship between competence satisfaction and concentration disruption. Finally, competence and relatedness satisfaction influenced the development of reflection directly and positively and, exclusively, relatedness satisfaction had a negative influence in a direct way on concentration disruption. In sum, our results highlight that (a) the environment of young footballers through psychological needs satisfaction has a positive (i.e., reflection) or negative (i.e., concentration disruption) influence on the field, but only with the mediation of harmonious passion, (b) in a competitive environment, the perception of competence can have a positive influence on concentration disruption, but only with the development of obsessive passion as a mediator, and (c) relatedness satisfaction plays a key role in distinguishing between reflection and concentration disruption.

## Introduction

The young athlete’s development could be based on, at least, two desired goals: continue improving sports skills and becoming elite athletes. However, becoming an elite athlete is one of the more difficult challenges that young athletes must cope with in their career development. Specifically, in football, Haugaasen and Jordet (2012) suggested that out of the 265 million people who regularly play football, only 0.04% will play in a professional league. Moreover, Green (2009) reports that out of 10,000 youngsters who play in the lower categories of professional English clubs each season, less than 1% will become professional players. These data show how difficult it is to become a professional player.

This study is contextualized in the realm of elite U18 male football players. This category could be considered as the last step before jumping to professional football (Chamorro et al., 2016). According to studies with this type of population (Mills et al., 2012; Drew et al., 2019), motivational variables, as well as cognitive skills, have a key role in the development of young athletes who practice sports. Exploring the relationships among motivational variables [i.e.. basic psychological needs (BPN) and passion] and cognitive skills (i.e., reflective thinking and concentration disruption) could help us to understand whether the influence of motivational factors in cognitive variables has an impact on the player’s development.

One of the macro-theories of motivation more used in sports has been the Self-Determination Theory (SDT; Deci and Ryan, 1985; Ryan and Deci, 2000; Clancy et al., 2017). According to SDT, people engage in various activities throughout life in the hope of satisfying BPN for autonomy (the need to experience volition, self-direction, and choice), competence (the need to feel a sense of efficacy), and relatedness (the need to feel loved, valued, and connected with significant others). SDT proposes that when an individual has satisfied their BPNs, he or she should experience optimal levels of physical and psychological health. In the field of physical activity and sports, research has shown that the satisfaction of the three BPNs has been associated with a wide range of positive outcomes, while the thwarting or non-satisfaction of BPNs has been linked to negative outcomes (Gunnell et al., 2013; Fernández-Ríos et al., 2014; Pulido et al., 2018, 2020; Sheehan et al., 2018).

According to Vallerand et al. (2003), the satisfaction of psychological needs contributes to better value an activity with which an individual has committed. This assessment of the activity (i.e., football) could lead to the development of passion for the activity (Vallerand, 2015). The Dualistic Model of Passion (DMP; Vallerand et al., 2003; Vallerand, 2010, 2015) proposes that it is important to differentiate the passion not only in terms of quantity, but also in terms of quality. Vallerand et al. (2003) distinguish two types of passion, obsessive and harmonious, in terms of how the passionate activity is regulated and integrated with other life domains. Obsessive passion (OP) refers to a strong and uncontrollable urge to partake in the activity. In this type of passion, the process of internalization of the activity into the identity of an individual occurs in a controlled manner, originating from intrapersonal and/or interpersonal pressure caused by contingencies associated with the activity (e.g., feelings of social acceptance or self-esteem). Harmonious passion (HP) also prompts a strong desire to engage in the activity as OP. However, HP refers to a strong desire to freely engage in the activity without any type of contingency, wherein the person regards the activity as a significant (but not overwhelming) part of his/her identity. With HP, the person is in control of the activity and freely decides whether or not to engage in the activity under given circumstances. According to the meta-analytical review by Curran et al. (2015), HP is positively related with adaptive intrapersonal outcomes (e.g., life satisfaction, flow, concentration, and vitality), and OP shows positive relationships with adaptive and less adaptive intrapersonal outcomes (e.g., mastery approach goals, burnout, rumination, and negative affects).

Theoretically, it could be assumed that BPN satisfaction promotes a better valuation for an activity and, consequently, more probabilities to develop ongoing passion for this activity in the future. Although the association between BPN and passion has already been investigated (Forest et al., 2011; Curran et al., 2013; Verner-Filion et al., 2017; Verner-Filion and Vallerand, 2018), only a few studies have tested the role of BPN satisfaction as an antecedent of passion (Alcaraz-Ibañez et al., 2016; Lalande et al., 2017; Tóth-Király et al., 2019). Studies by Lalande et al. (2017) suggested that the satisfaction of BPNs determines the type of passion that an individual experiences toward an activity, even when he or she is already passionate about it. In that study, BPN satisfaction inside of passionate activity was found as a significant predictor of both HP and OP, although the effect was stronger for HP. In the same way, Alcaraz-Ibañez et al. (2016) found that BPN satisfaction was a significant predictor of both types of passion for exercise, and again the effect was strong for HP. In the study by Tóth-Király et al. (2019) with screen-based leisure activities, they assessed BPN satisfaction in life, in general, (not inside of passionate activity) and showed that general needs satisfaction negatively predicted OP but had no relation to HP. However, it should be noted that these previous studies did not address BPN satisfaction as three separate factors. Thus, the influence of each BPN satisfaction on passion inside of passionate activity has not been explored enough.

On the other hand, Pierce (2018) comments that young people learn cognitively, as well as acquire behavioral, intrapersonal, and interpersonal skills through sports and put themselves in an excellent position to be better athletes. In this sense, the athlete’s development and, therefore, the development of future elite athletes, involves, in some way, a continuous learning process that can allow improvements in the development of the skills of the sport. Ntoumanis and Cumming (2016) point out that self-regulation has been identified as fundamental to successful learning, well-being, and performance and, thus, enables athletes to realize their potential in sports as well as other life domains. Self-regulated learning implies that individuals assume their own learning tasks proactively from metacognitive and motivational strategies (Zimmerman, 2008; Bartels and Magun-Jackson, 2009). Within self-regulated learning (Zimmerman, 2006), reflection (i.e., the extent to which players reappraise what they have learnt and adapted past knowledge and experience to improve performance) seems to play a significant role in talent, improvement, and performance (Toering et al., 2009; Gledhill and Harwood, 2014; Gledhill et al., 2017; Jonker et al., 2019). Jonker et al. (2019) showed that elite young players with high scores on reflective thinking (as a self-regulatory skill) were more likely to develop and, thus, reach the level of professional football. This study found that players who achieved professional football status increased their levels of reflection in the process of transitioning to professional clubs, while reflection levels in semi-professional or non-professional players remained stable. Thus, reflection could be considered as an adaptive cognitive skill regarding an athlete’s development that needs the promotion of metacognitive and motivational strategies (Zimmerman, 2008; Bartels and Magun-Jackson, 2009).

In this sense, it could be inferred that, for a process of reflection on a given action or set of actions to be more likely, the athlete should be focused on such action. Consequently, it might not be compatible with a reflective process to occur after an action in which it has been deconcentrated. Concentration disruption was defined by Grossbard et al. (2009) as the athlete’s difficulty in focusing on the key aspects of the task to be performed that impedes clarity of thought during the competitive situation. In the sporting context, concentration disruption and worry were included by Smith et al. (1990) as a cognitive component of pre-competitive anxiety. Unlike reflection, concentration disruption has been linked to undesired variables for athletes. McCarthy et al. (2013) suggest that concentration disruption in young athletes is related to displeasing emotions such as dejection, anxiety, and interfering thoughts. Thus, concentration disruption could be considered as a maladaptive cognitive skill with a negative influence on an athlete’s development.

Motivational variables and cognitive skills are fundamental in the promotion of an athlete’s development (Knight et al., 2019). Among adaptive cognitive skills, reflective thinking as a component of self-regulated learning has been identified as a key variable of an athlete’s development and sports performance. Successful self-regulated learnings occur when individuals are motivated and determined to use their cognitive and metacognitive strategies (Bilde et al., 2011). In fact, more determined forms of motivation have been related to higher levels of self-regulated learning (Bilde et al., 2011). Motivational variables, such as psychological needs satisfaction and passion, could be candidates to precede the self-regulated learning and, thus, also reflective thinking. In previous studies, psychological needs and passion have also been identified as antecedents of other adaptive cognitive skills, such as goal-setting experiences, concentration, flow, or rumination (Curran et al., 2015; Gledhill et al., 2017). In addition, the inclusion of both factors in a model testing the mediator role of types of passion between the influence of each BPN satisfaction as antecedents on cognitive skills (i.e., refection and concentration disruption) has not been explored enough. Following other studies (Alcaraz-Ibañez et al., 2016; Lalande et al., 2017) and understanding that the satisfaction of BPNs is necessary for the evaluation of the activity and the subsequent development or maintenance of both types of passion (Vallerand, 2015), this work expects types of passion to play a mediating role between BPN satisfaction and cognitive skills. In this study, we also wanted to extend the previous literature identifying antecedents of reflective thinking, because self-regulation and its elements have received less research attention in sports compared to other contexts such as health or education (Ntoumanis and Cumming, 2016).

Thus, the aim of this study is to explore the influence of each BPN satisfaction on players’ development through reflection and concentration disruption with the mediation of types of passion in Spanish young elite football players. Based on the aforementioned literature, we hypothesize that each psychological needs satisfaction would predict positively and directly reflection, and positively with the mediation of HP. In turn, each psychological needs satisfaction would predict negatively and directly concentration disruption, and also negatively with the mediation of HP. Also, we hypothesize that OP would facilitate concentration disruption but, due to controversial findings regarding the influence of satisfaction of BPNs on OP, no hypothesis was established about the mediator role of OP in the model.

## Materials and Methods

### Participants

The elite young football players who are in the position, where they have to cope with the transition from junior to senior in a short time is a specific population within the practice of youth sports. For this reason, the main selection criterion of the participants was players who belonged to Spanish teams of Liga División de Honor Juvenil, considered as the last category before jumping into professional football. Specifically, players who belonged to clubs whose senior teams played in the Spanish First or Second Division were eligible. Finally, study participants were 478 young male elite football players (*M_age_* 17.42, *SD* = 0.705, range = 16–19) of 27 academy teams of the highest Under-18 category in Spain. This category is divided into seven competition groups with 16 teams, each classified by the geographical regions of Spain. The teams assessed in the study compete in 6 of the 7 groups, with one exception from the Canary Islands group. It is also necessary to point out that all the teams that participated in the study finished the league within the top six rankings of their groups.

### Measures

All questionnaires were administered in the Spanish language. Coefficients Omega (McDonald, 1970) for each scale are shown in Table 1.

**Table 1:** Descriptive statistics, omega coefficients and disattenuated correlations among variables measures in the study.

Scale	Range	*M* (*SD*)	1	2	3	4	5	6	7
1. Autonomy satisfaction	1–7	4.95 (1.13)	*0.73*						
2. Competence satisfaction	1–7	5.45 (0.91)	0.27^*^	*0.84*					
3. Relatedness satisfaction	1–7	5.85 (1.14)	0.39^**^	0.30^**^	*0.90*				
4. Harmonious passion	1–7	5.94 (0.86)	0.34^**^	0.42^**^	0.52^**^	*0.83*			
5. Obsessive passion	1–7	4.33 (1.33)	0.02	0.21^*^	0.01	0.41^**^	*0.80*		
6. Reflection	1–7	6.04 (0.82)	0.31^**^	0.37^**^	0.45^**^	0.68^**^	0.26^*^	*0.87*	
7. Concentration disruption	1–4	1.40 (0.43)	−0.01	-0.20^*^	−0.39^**^	−0.34^**^	0.26^*^	−0.32^**^	*0.77*

The table presents correlations between factors (i.e., disattenuated correlations). Omega coefficients are listed in italics in the diagonal.

* *p* < 0.01;

** *p* < 0.001.

Satisfaction of BPN was measured using Spanish adaptions of the (a) autonomy satisfaction scale by Standage et al. (2005) and Alcaraz et al. (2013), (b) competence satisfaction by McAuley et al. (1989) and Balaguer et al. (2008), and (c) the relatedness satisfaction by Richer and Vallerand (1998) and Balaguer et al. (2008). Following the stem “In soccer…” five items measured autonomy satisfaction (i.e., “I can decide which activities I want to practice”) and relatedness satisfaction (i.e., “I feel understood”) and six items measured competence satisfaction (i.e., “I think I am pretty good at soccer”). The three scales were assessed on a 7-point Likert scale (1 = do not agree at all, 7 = very strongly agree). Previous research has shown that these scales possess adequate internal structure and internal consistency (Standage et al., 2005).

A Spanish version (Chamarro et al., 2015) of the Passion Scale (adapted for soccer; Marsh et al., 2013b) with six items measuring HP (e.g., football allows me to live a variety of experiences) and six items measuring OP (e.g., I cannot live without soccer) was applied to measure the athletes’ passion for soccer. Also, general passion for football in each participant was assessed using five items of the general passion criteria (e.g., “This activity is important for me”). The scale was assessed on a 7-point Likert scale (1 = do not agree at all, 7 = very strongly agree).

A Spanish version of the 5-item Reflective Learning Continuum (RLC), developed by Peltier et al. (2006) and adapted to football (Toering et al., 2013), was used to measure the extent to which respondents could appraise what they have learned and adapted their past knowledge and experiences to improve performance. An example question is “I often reappraise my experiences so I can learn from them.” Items were rated on a 7-point Likert-type scale ranging from 1 (strongly disagree) to 7 (strongly agree).

A Spanish version of the concentration disruption subscale of the Sport Anxiety Scale-2 by Smith et al. (2006) and Ramis et al. (2010) was used to assess the degree to which participants experienced concentration disruption before and during sports competitions. Participants were instructed to respond to each of the five items (e.g., “It is hard for me to focus on what I am supposed to do”), concerning how they felt during their most recent sporting encounter on a 4-point scale (1 = not at all, 2 = a little, 3 = pretty much, and 4 = very much).

### Procedures

First, ethical approval for the study was granted by the authors’ university Ethics Committee. At the beginning of the investigation, clubs were contacted by phone and the study and its objectives were explained. Once they agreed to participate, the dates were determined for the first author to travel to all clubs within a short period of time. All clubs were visited and surveyed within 1 month. The measuring occurred at the end of the season. With the permission of each youth academy manager and each coach, all participants attended and voluntarily participated in the study. They were informed about the objectives, guaranteed anonymity, and confidentiality and gave informed consent. All participants signed a consent form in which they were informed that they participated voluntarily and that their data were confidential.

### Data Analysis

#### Preliminary Analysis and Measurement Models

Preliminary analyses included the study of missing values, data distribution, and scale internal consistency. In order to identify the general passion of participants, the data of passion criteria were included. Using as reference other works (e.g., Moeller et al., 2015; Vallerand, 2015), we used the midpoint of the response scale in the general passion criteria score to distinguish passionate individuals (i.e., mean score 4 or higher) from non-passionate individuals (i.e., mean score below 4). In addition, all measurement models were estimated using the weighted least squares means and variance (WLSMV) adjusted estimator with the MPLUS 7.0 software, because this estimator is adequate for categorical data (Muthén and Muthén, 2012). Model fit was assessed with the fit indexes *χ* ^2^/gl root mean square error of approximation (RMSEA), comparative fit index (CFI), Tucker-Lewis Index (TLI), and standardized root mean squared residual (SRMR). Regarding the χ^2^/gl values, ratios from 2 to 1 and 3 to 1 are indicative of an acceptable fit to the data (Carmines and McIver, 1981). Furthermore, based on the criterion of Hu and Bentler (1999), CFI and TLI values >0.95 and RMSEA <0.06 are seen as indicative of an excellent fit to the data. CFI y TLI values >0.90 and SRMR and RMSEA <0.08 are seen as indicators of acceptable fit (Marsh et al., 2004; Kline, 2011).

On the one hand, to evaluate the factorial structure of the questionnaires in which only one factor was expected (reflection and concentration), a confirmatory factor analysis (CFA) model was tested. On the other hand, to evaluate the factorial structure of those questionnaires with more than one expected factor (BPN satisfaction and passion), an exploratory structural equation model (ESEM) was tested. This decision was made following the suggestion of Marsh et al. (2013a) to use the model with the best psychometric properties according to the fit indices of each model and the interpretability of the factors obtained (Alcaraz et al., 2015). Furthermore, the use of ESEM as confirmatory structural analysis is at least as optimal as the use of CFA based on strong theoretical assumptions regarding the expected factor structure (Guay et al., 2015).

#### Mediation Models

The analyses of the mediation models were also conducted using MPLUS 7.0 software (Muthén and Muthén, 2012). Before testing the models, the disattenuated correlations between factors were estimated according to the measurement models obtained previously. Also, the descriptive statistics and the coefficient omega of all variables were estimated. Correlation coefficients were interpreted following the criteria of Safrit and Wood (1995) in terms of non-correlation (0–0.19), low correlation (0.20–0.39), moderate correlation (0.40–0.59), moderate to high correlation (0.60–0.79), and high correlation (≥0.80). Next, it was explored how HP and OP mediated the relationships of autonomy (A), competence (C), relatedness (R), satisfaction, and reflection (RE) and concentration disruption (CD). According to MacKinnon (2008), a mediating variable “is intermediate in the causal path from an independent variable to a dependent variable” (p. 8). The possibility of mediation was tested using three different models (see Alcaraz et al., 2015). The first model (i.e., Model Partial Mediation; MPM) was tested for partial mediation (i.e., direct and indirect effects), the second model (i.e., Model Complete Mediation; MCM) was tested for complete mediation (i.e., only indirect effects), and the third model (i.e., Model Direct Effects; MDE) was tested for the absence of mediation (i.e., only direct effects). We hypothesized that relations between athletes’ BPN satisfaction and reflection and concentration disruption would be partially mediated by HP and OP.

We tested all three structural equation models using the WLSMV estimator for categorical data. Following the guidelines of Marsh et al. (2013a), the comparison between models was based on a chi-square difference test and CFI, TLI, and RMSEA differences. Although chi-square is sensitive to sample size, some authors have suggested that the more parsimonious model can be accepted if it presents equal or better fit indexes as the more restrictive model (Chen, 2007; Marsh et al., 2013a; Ramis et al., 2017). The factors included in all three structural models were defined according to the measurement models described in the previous step of our data analysis. Mediated effects were obtained using the Model Indirect command and the *VIA* instruction under the Delta parameterization as defined in MPLUS.

## Results

### Preliminary Analysis and Measurement Models

First, the data distribution was explored. Overall, data tended to demonstrate high values for autonomy, competence, and relatedness satisfaction, both types of passion, and reflection (i.e., ceiling effects). In contrast, concentration disruption values tended to be low (i.e., floor effect). Also, the levels of skewness (−1.94 to 2.05) and kurtosis (−0.98 to 5.20) were explored. These results suggested the non-normality of the observed data and, consequently, were treated as categorical data in subsequent analyses (Alcaraz et al., 2015). Regarding the general passion of participants, only three of them scored below 4 points. Thus, 99.4% of the participants were considered as passionate for football. Second, the measurement models for each instrument in the study were analyzed. Regarding instruments with a single-factor structure, measurement models showed good fit for reflection except for RMSEA: *X* ^2^ (df) = 28.609 (5), *p* < 0.001, RMSEA = 0.099 (90% CI [0.066, 0.136]), CFI = 0.993, TLI = 0.986, SRMR = 0.062; and excellent fit for concentration disruption: *X* ^2^ (df) = 7.097 (5), *p* = 0.001, RMSEA = 0.030 (90% CI [0.000, 0.075]), CFI = 0.998, TLI = 0.996, SRMR = 0.035. For instruments whose structure was made up of more than one factor, an ESEM approach was used. In this way, the measurement models showed acceptable fit for passion: *X* ^2^ (df) = 351.340 (72), *p* < 0.001, RMSEA = 0.080 (90% CI [0.111, 0.135]), CFI = 0.938, TLI = 0.905, SRMR = 0.059; and for BPN satisfaction: *X* ^2^ (df) = 345.958 (75), p < 0.001, RMSEA = 0.079 (90% CI [0.018, 0.96]), CFI = 0.967, TLI = 0.947, SRMR = 0.058.

### Mediation Model

Table 1 shows the correlations between factors obtained with the measurement models mentioned above (i.e., disattenuated correlations) as well as the mean, standard deviations, and coefficient omega of the study factors. Except for OP-autonomy/relatedness satisfaction and autonomy satisfaction-concentration disruption, all correlations were significant. Both PA and reflection showed low and moderate positive correlations with all study variables except concentration disruption, whose correlations were low and negative. OP correlated positively with both competence satisfaction and reflection and, concentration disruption. The other correlations were consistent with our expectations. Also, coefficient omega (*ω*) of McDonald (1970) ranged from 0.73 to 0.90.

Both Model Partial Mediation (MPM) and Model Complete Mediation (MCM) exhibited excellent fit to the data (see Table 2). However, as shown in Table 2, MPM fit slightly better than MCM (*X* ^2^ (df) = 1144.309 (608), *p* < 0.001, RMSEA = 0.043 (90% CI [0.039, 0.047]), CFI = 0.962, TLI = 0.956, SRMR = 0.038), and therefore the partial mediation role of type of passion in the paths from BPN satisfaction to concentration disruption and reflection was supported.

**Table 2:** Fit statistics and standardized coefficient estimates for mediation structural models.

Model	*X* ^2^ (df)	*X* ^2^/df	RMSEA (CI90)	CFI	TLI	Δ*χ* ^2^ (Δdf)	ΔRMSEA	ΔCFI	ΔTLI
1. Model Partial med (MPM)	1144.309* (608)	1.882	0.043 (0.039–0.047)	0.962	0.956				
2. Model Complete med (MCD)	1119.766* (614)	1.824	0.045 (0.041–0.048)	0.959	0.953	34.877* (6)	0.002	−0.003	−0.003
3. Model Direct eff (MDE)	1815.335* (612)	2.966	0.064 (0.061–0.068)	0.915	0.903	197.707* (4)	0.021	−0.047	−0.053

1. Model Partial Med: Model 1, partial mediation model; 2. Model Complete Med: Model 2, complete mediation model; 3. Model Direct Eff: Model 3, direct effects model. *p < .05.

**Figure 1:**
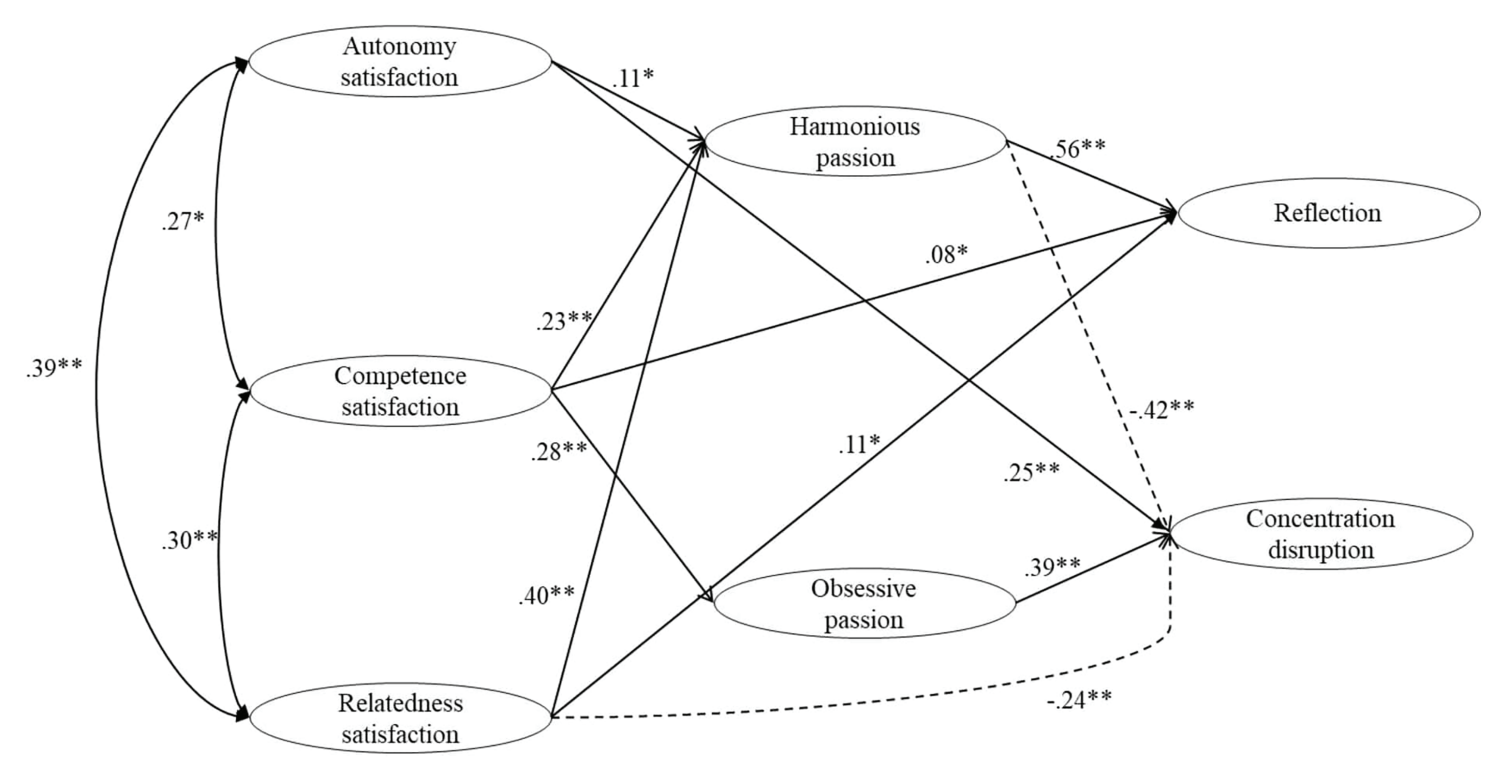
Structural equation model MPM presenting partial mediation of players’ types of passion in the relationship between satisfaction psychological needs, and reflection and concentration disruption. *Note.* For presentation simplicity purposes, only standardized statistically-significant paths. Dashed lines indicate negative paths. ^*^ *p < 0.05*; ^**^ *p < 0.01*.

In Figure 1, we present the results of the MPM and total and indirect effects for the MPM are presented in Table 3. The amount of variance explained in MPM is R^2^_Concentration disruption_ = 0.34, R^2^_Reflection_ = 0.48.

**Table 3:** Standardized total and indirect effects for the model partial mediation.

	Total effects		Indirect effects	
	Estimate	95% CI	Estimate	95% CI
Autonomy → reflection	0.063^*^	0.006 to 0.119		
*Harmonious passion*			0.063^*^	0.006 to 0.119
*Obsessive passion*			0.000	−0.003 to 0.002
Competence → reflection	0.158^**^	0.104 to 0.211		
*Harmonious passion*			0.156^**^	0.099 to 0.213
*Obsessive passion*			0.002	−0.021 to 0.025
Relatedness → reflection	0.219^**^	0.148 to 0.291		
*Harmonious passion*			0.219^**^	0.150 to 0.289
*Obsessive passion*			0.000	−0.005 to 0.005
Autonomy → CD	−0.056^*^	−0.108 to −0.004		
*Harmonious passion*			−0.047^*^	−0.091 to −0.003
*Obsessive passion*			−0.009	−0.053 to 0.035
Competence → CD	−0.025	−0.082 to 0.031		
*Harmonious passion*			−0.117^**^	−0.172 to −0.061
*Obsessive passion*			0.091^**^	0.040 to 0.143
Relatedness → CD	−0.184^**^	−0.254 to −0.113		
*Harmonious passion*			−0.164^**^	−0.233 to −0.096
*Obsessive passion*			−0.019	−0.062 to 0.024

CD, concentration disruption; CI, confidence intervals. Italics refer to indirect effects of the model.

* *p* < 0.05;

** *p* < 0.01.

Regarding the significant effects of MPM, we observed that (a) satisfaction of players’ autonomy was indirectly and positively related to reflection (A → HP → RE [95% CI] = 0.06 [0.006, 0.119]) and indirectly and negatively to concentration disruption (A → HP → CD [95% CI] = −0.05 [−0.091, −0.003]), both through HP, and directly and positively related to concentration disruption (A → CD = 0.25), (b) satisfaction of need for competence presented an indirect positive effect on reflection (C → HP → RE [95% CI] = 0.16 [0.099, 0.213]) and indirect negative effect on concentration disruption (C → HP → CD [95% CI] = −0.18 [−0.712, −0.061]), both through HP, a positive indirect effect on concentration disruption through OP (C → OP → CD [95% CI] = 0.09 [0.040, 0.143]) and a positive direct effect on reflection (C → RE = 0.08), and (c) satisfaction of players’ relatedness showed a negative indirect positive effect on reflection (R → HP → RE [95% CI] = 0.22 [0.150, 0.289]) and an indirect negative effect on concentration disruption (C → HP → CD [95% CI] = −0.16 [−0.233, −0.096]), both through HP, a positive direct effect on reflection (R → RE = 0.11) and a negative direct effect on concentration disruption (R → CD = −0.24).

## Discussion

This study aimed to explore the influence of each BPN satisfaction on player’s development through reflection and concentration disruption with the mediation of types of passion in Spanish young elite football players. The results provide a support for the partial mediation model, where each psychological needs satisfaction prompt reflection and concentration disruption with the mediation of types of passion. Specific findings show that psychological needs satisfaction has a positive influence on reflection and a negative influence on concentration disruption, both with the mediation of HP. In addition, OP mediated the positive relationship between competence satisfaction and concentration disruption. Finally, competence and relatedness satisfaction influence the development of reflective thinking directly and positively, and relatedness satisfaction has a negative influence directly on concentration disruption. Reflective thinking as an adaptive cognitive skill (Ntoumanis and Cumming, 2016) was hypothesized as incompatible with concentration disruption and the negative correlation between reflection and concentration disruption could partially support this assumption.

The findings also support the main hypothesis that the mediation of HP discriminates the kind of partial relationship that is established among the influence of each BPN satisfaction on reflection (i.e., positive) and concentration disruption (i.e., negative). These results agree with DMP (Vallerand, 2015), which postulates that the quality of passion influences how cognitive processes occur. DMP proposes that with HP, adaptive integrative self-processes are at play, leading the person to fully partake in the activity with an openness that is conducive to mindful attention, concentration, and flow. Instead, with OP ego-invested processes are involved and such processes lead individuals to share task attention with other external elements, such as the outcomes and other participants. Thus, OP should lead to cognitive processes of lesser quality than those that originate from HP, in our findings, prompting concentration disruption. In the meta-analytical review of passion developed by Curran et al. (2015), HP also showed a positive influence in adaptive cognitive skill as concentration or flow and as a negative influence with anxiety or rumination. Conversely, in this study OP showed a positive influence on less or maladaptive cognitive skills as rumination and anxiety.

Ertmer and Newby (1996) identified reflection as the key process to expert learning, due to the translation of knowledge into action, making it possible to gain strategy knowledge from specific activities. According to the existing literature (Toering et al., 2009; Gledhill and Harwood, 2014; Gledhill et al., 2017; Jonker et al., 2019), reflective thinking is desirable in an athlete’s development. For example, Jonker et al. (2019) showed that elite young players with higher values toward reflective thinking were more likely to succeed and, also, Toering et al. (2009) found that a high score in reflection seemed to be associated with a high-performance level. Theoretical assumptions as well as other studies have proposed that motivation is essential to develop self-regulation learning and reflective thinking in individuals (Zimmerman, 2006, 2008; Bartels and Magun-Jackson, 2009; Bilde et al., 2011). Within motivation, our findings support the role of BPN satisfaction and types of passion in prompting reflective thinking and protecting against other less desirable cognitive processes, such as concentration disruption. Thus, the development of HP through an athlete’s perception of autonomy, competence, and relatedness supported by the environment, should facilitate a positive athlete’s development.

In this line, it was OP, rather than HP, that mediated the relationship between competence satisfaction and concentration disruption. The environment of the sample used in this study is extremely competitive, characterized by internal (i.e., beliefs about the self-performance and performance compared to other teammates and do not disappoint the significant others) and external pressures (i.e., family, coaches, and organizations) to reach senior levels (Drew et al., 2019) and performance. If a young football player feels competent, he may feel more pressure to be better than his teammates and not disappoint his nearest environment (i.e., coach, family, and friends). These reasons are more controlled than autonomous, and he could develop an ego-invested process, more characteristic of OP than HP (Vallerand, 2015). Later, OP prompts the cognitive skill-less adaptive, in this case, concentration disruption (Curran et al., 2015). In addition, it seems that BPNs satisfaction (i.e., with the exception of relatedness satisfaction) does not have a negative influence on concentration disruption through OP either. In other contexts, other studies have suggested that it is possible that, while psychological needs satisfaction facilitates HP, needs frustration would imply an impact on the development of OP (Alcaraz-Ibañez et al., 2016; Tóth-Király et al., 2019). In any case, future studies should be addressing this relationship in young athletes.

Moreover, relatedness satisfaction was the only BPN satisfaction that discriminated directly between a positive influence in reflective thinking and a negative influence in concentration disruption. According to our results, to feel loved, valued, and connected with significant others in a passionate activity influences the quality of the cognitive process. In this sense, the importance of relatedness satisfaction as a strong predictor of positive intrapersonal outcomes (i.e., positive affect or subjective vitality) has been stated in past research (Alcaraz et al., 2015; Perez-Rivases et al., 2017).

### Limitation, Future Research, and Implications

Although the present study yields important findings, certain limitations and avenues for future research need to be considered. First, only elite U18 male football players were studied in the present research. Future studies should include the model proposed in other sports, competitive levels, and female athletes. Second, although all the teams assessed had similar competitive characteristic (i.e., club level, competitive level, and the final rank of the team), some variables such as games played of each player could have a moderator effect. Third, it is highly recommended to carry out longitudinal research to analyze how these variables and the relationship between them has developed during the whole season. Fourth, a model with the BPN thwarting or behavioral regulations, as a level more inside of the proposed model, could be interesting.

This research is contextualized in elite U18 male football players, considered as the last step before jumping to professional football (Chamorro et al., 2016). Drew et al. (2019) showed that motivational variables, as well as cognitive skills, facilitate the athlete’s development and their progression to professional sports. Our results can help practitioners take into account several issues when working with young athletes who are in a competitive phase of their athletic careers. Also, the results of our study support the role of motivational variables to promote cognitive processes in young elite athletes. Those results have implications for the athletes’ environment on developing a healthier passion for sport and the use of more adaptive cognitive strategies for athletes. For example, coaches or coaching staff are essential in supporting the BPNs of athletes. Previous studies have identified strategies that help coaches to support athletes’ BPNs (Hancox et al., 2018; Hook and Newland, 2018). The implementation of these strategies would have an impact on the development of HP, the promotion of the athlete’s reflective thinking and, consequently, promoting the athlete’s positive development.

## Conclusion

The athlete’s development involves a continuous learning process of skills in sport. Enhancing reflection and preventing concentration disruption can have a positive effect in that continuous learning process. With a sample of elite young football players, this study focuses on how motivational variables (i.e., BPN satisfaction and passion) can influence the development of those cognitive skill simplicated in athlete’s development. Our findings show that the environment of young players, through to BPN satisfaction, and the development or ongoing of HP for football has a positive influence in the development of reflective thinking and a negative influence on concentration disruption. Among BPN satisfaction, in particular relatedness satisfaction plays a key role in distinguishing between reflective thinking and concentration disruption. Practitioners should monitor the competence satisfaction in a competitive environment, due to it would be possible that it had a positive impact on maladaptive cognitive variables (i.e., concentration disruption) along with OP.

## Data Availability Statement

The raw data supporting the conclusions of this article will be made available by the authors, without undue reservation.

## Ethics Statement

The studies involving human participants were reviewed and approved by Comisión de investigación Universidad Europea de Madrid. Written informed consent to participate in this study was provided by the participants’ legal guardian/next of kin.

## Author Contributions

JC contributed to this work following these tasks: conceptualization, data curation, formal analysis, investigation, methodology, writing – original draft, and writing – review and editing. RM contributed to this work following these tasks: conceptualization, formal analysis, investigation, methodology, writing – original draft, and writing – review and editing. TG-C contributed to this work following these tasks: data curation, formal analysis, investigation, methodology, and writing – review and editing. MT contributed to this work following these tasks: conceptualization, data curation, formal analysis, investigation, methodology, visualization, and writing – review and editing. All authors contributed to the article and approved the submitted version.

### Conflict of Interest

The authors declare that the research was conducted in the absence of any commercial or financial relationships that could be construed as a potential conflict of interest.
